# Reduced soluble RAGE is associated with disease severity of axonal Guillain-Barré syndrome

**DOI:** 10.1038/srep21890

**Published:** 2016-02-23

**Authors:** Da-Qi Zhang, Rong Wang, Ting Li, Jian-Ping Zhou, Guo-Qiang Chang, Ning Zhao, Li-Na Yang, Hui Zhai, Li Yang

**Affiliations:** 1 Department of Neurology and Tianjin Neurological Institute, Tianjin Medical University General Hospital, Tianjin 300052, China; 2 School of Integrative Medicine, Tianjin University of Traditional Chinese Medicine, Tianjin 300193, China

## Abstract

Soluble receptor for advanced glycation end products (sRAGE) is an anti-inflammatory factor that mitigates the proinflammatory effects of high mobility group box 1 (HMGB1). The aim of this study was to investigate whether Guillain-Barré syndrome (GBS)-related inflammation are mediated by sRAGE and HMGB1. We measured serum sRAGE, HMGB1, IL-6, and TNF-α levels in 86 patients with GBS and analysed associations between sRAGE or HMGB1 and clinical variables in these subjects. In addition, we determined cerebrospinal fluid sRAGE and HMGB1 levels in a cross-sectional study of 50 patients with GBS who had matched serum samples. We found serum sRAGE levels in patients with the acute motor axonal neuropathy (AMAN) subtype of GBS, but not other subtypes, were significantly lower than those in healthy controls, and were significantly correlated with GBS disability score and Erasmus GBS outcome score, while serum HMGB1, IL-6, and TNF-α levels in all subtypes of GBS were significantly higher than those in healthy controls. Moreover, increased sRAGE levels and decreased HMGB1 levels after treatment were observed. Our results showed that serum sRAGE may be a useful biomarker for inflammation in the AMAN GBS subtype, while HMGB1 may be related to the inflammatory process across all types of GBS.

Guillain-Barré syndrome (GBS) is an acute, post-infectious, immune-mediated spectrum disorder that affects the peripheral nervous system. A series of epidemiological studies have discerned that the two most common GBS subtypes differ in their geographical distributions: acute inflammatory demyelinating polyneuropathy (AIDP) is the dominant subtype in North America, Europe, and Australia, while in Asia, especially in China, the proportion of acute motor axonal neuropathy (AMAN) in GBS is approximately 30–65%^1^,^2^,^3^,^4^,^5^. Unlike AIDP, AMAN is correlated with preceding Campylobacter jejuni infection and the presence of antibodies to gangliosides. Furthermore, AMAN may present with a more severe clinical course and poorer outcome than AIDP. Notably, AMAN can sometimes simultaneously involve sensory peripheral nerves, a condition termed acute motor and sensory axonal neuropathy (AMSAN)^6^. Taken together, the different geographical distributions and clinical manifestations of AIDP versus AMAN suggest that the two subtypes of GBS could have different immunopathogenic mechanisms.

However, differences in the immunologic profiles that might distinguish GBS subtypes and explain their different pathologies are poorly understood. Among the immunologic factors heavily implicated in autoimmune disorders is the receptor for advanced glycation end products (RAGE), a 35-kDa multi-ligand glycoprotein belonging to the immunoglobulin superfamily^7^. RAGE, though first described as a receptor for advanced glycation end products (AGEs), can bind to several endogenous ligands such as high mobility group box 1 (HMGB1), S100/calgranulins and β-amyloid; thus RAGE is a potent mediator of inflammation^8^. *In vivo*, human RAGE is expressed as several different isoforms: a full-length membrane-bound RAGE(mRAGE) which consists of an extracellular, transmembrane, and cytosolic domain; a N-truncated RAGE and a C-truncated RAGE without a transmembrane and cytosolic domain. The latter of these, which can be released extracellularly, is also called soluble RAGE (sRAGE)^9^. Plasma sRAGE is formed either by alternative splicing of RAGE mRNA or by proteolytic cleavage of mRAGE protein^9^. Activation of mRAGE is involved in inflammatory-related processes in many types of cells, including endothelial cells, smooth muscle cells, monocytes, macrophages, and neurons^10^,^11^. By contrast, studies have shown that circulating sRAGE acts as a decoy that can compete with the mRAGE for ligand binding and then hinders mRAGE-related intracellular signaling^12^.

Moreover, recent evidence has indicated that HMGB1-mRAGE interactions play an essential role in the development and progression of immune-mediated disorders, such as rheumatoid arthritis, multiple sclerosis and neuromyelitis optica^13^,^14^,^15^,^16^. Among the many different extracellular ligands of RAGE, HMGB1 is a nuclear protein loosely bound to DNA that is located in nucleosomes and regulates gene transcription. HMGB1 can be secreted extracellularly during inflammation and neuronal injury^17^,^18^. In addition, HMGB1 can sustain inflammation by promoting the migration of inflammatory cells, trigger immune responses generated from the invasion of microorganisms, and cause neuronal cell death^19^,^20^. HMGB-1 is mainly secreted from activated macrophages, mature dendritic cells, and necrotic cells and results in increased production of cytokines such as TNF-α and IL-6, as well as induction of B-cell activation^21^,^22^,^23^,^24^.

The roles of sRAGE and HMGB1 in the inflammatory processes of GBS have not been conclusively identified. To investigate whether sRAGE or HMGB1 is involved in GBS-related inflammation in a general or subtype-specific manner, we determined their serum and CSF levels in subtypes of GBS. We extended this investigation by focusing on the possible relationships of these two inflammatory factors with disease severity and treatment parameters in subtypes of GBS.

## Results

### Demographic and clinical features of subjects

The presenting demographic and clinical features of the study subjects are summarized in Table 1. All the participants were Han Chinese. There were no differences in age and gender among the three subtypes of GBS. When comparing the GBS disability score and EGOS among the AIDP, AMAN, and unclassified patients with GBS, we found that the GBS disability scores and EGOS in AMAN were significantly higher than those in AIDP and the unclassified subtype (p < 0.001, respectively, Table 1). In addition, we observed significantly higher protein levels in AMAN than in AIDP and the unclassified subtype (p = 0.022, p = 0.013, respectively, Table 1).

### Decreased serum sRAGE levels in AMAN

**Figure 1:**
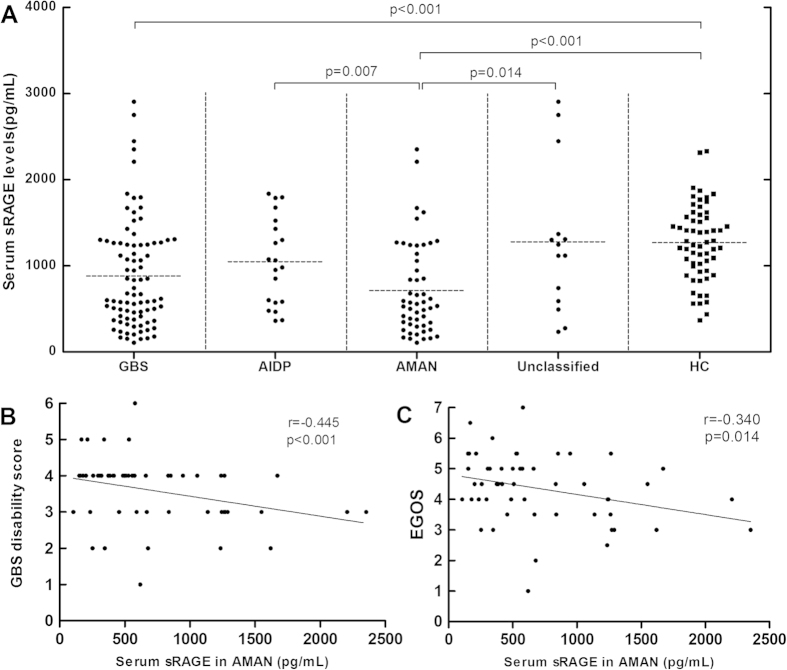
Serum soluble receptor for advanced glycation end products (sRAGE) levels in patients with Guillain-Barré syndrome (GBS). (**A**) Serum sRAGE levels in 86 patients with GBS – including 20 acute inflammatory demyelinating polyneuropathy (AIDP) subtype, 52 acute motor axonal neuropathy (AMAN) subtype, and 14 unclassified cases – and 60 healthy controls. (**B**) The relationship between serum sRAGE levels and GBS disability score in AMAN. (**C**) The relationship between serum sRAGE levels and Erasmus GBS outcome score (EGOS) in AMAN.

**Figure 2:**
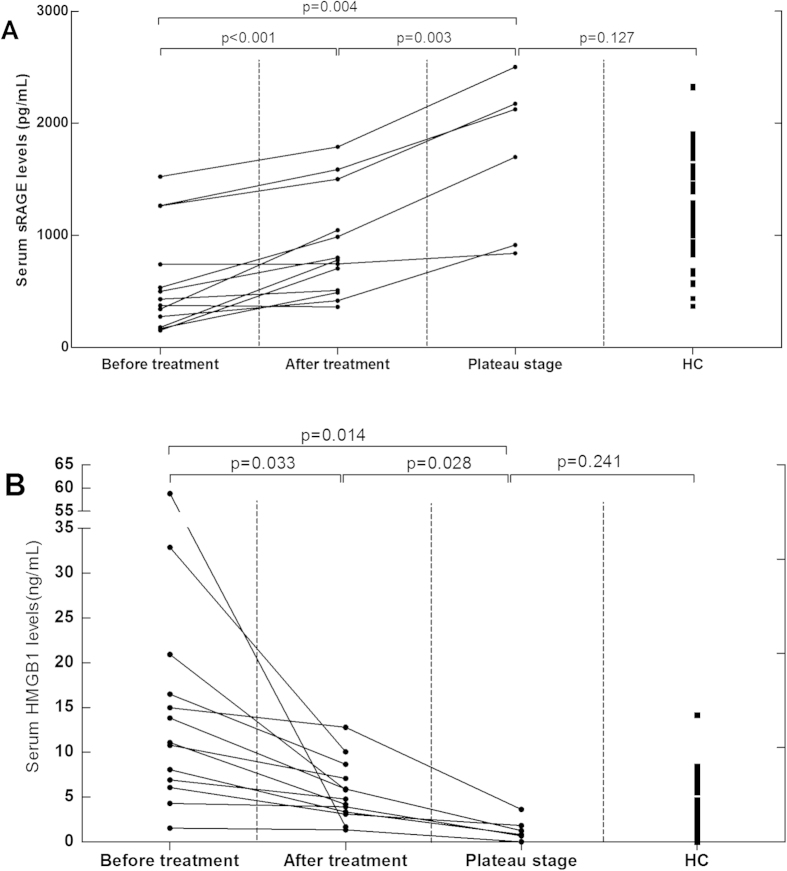
Serum soluble receptor for advanced glycation end products (sRAGE) and high mobility group box 1 (HMGB1) levels in patients with Guillain-Barré syndrome (GBS) before and after treatment. (**A**) Serum sRAGE levels in patients with GBS before treatment, after treatment, and in plateau phase. (**B**) Serum HMGB1 levels in patients with GBS before treatment, after treatment, and in plateau phase.

To investigate whether sRAGE correlates with GBS-mediated inflammation, we analysed sRAGE levels in patients with GBS and healthy controls. We found serum sRAGE levels were significantly decreased in GBS patients compared to controls (p < 0.001, Fig. 1A). Within GBS subtypes, we observed that sRAGE levels in patients with AMAN were significantly lower than those in patients with AIDP (p = 0.007, Fig. 1A) and unclassified GBS cases (p = 0.014, Fig. 1A), and control subjects (p < 0.001, Fig. 1A). Moreover, we found that the patients with AIDP had sRAGE levels similar to both unclassified patients (p = 0.612) and control subjects (p = 0.101). No difference was found between unclassified patients and healthy controls (p = 0.460). In the samples taken before and after treatment in 13 patients with GBS, serum sRAGE levels were significantly increased in patients after treatment (p < 0.001, Fig. 2A). Likewise, serum sRAGE levels in plateau phase were significantly increased compared with patients with GBS after treatment (p = 0.003, Fig. 2A). There was no significant difference in sRAGE levels between patients in plateau stage and healthy controls (p = 0.127, Fig. 2A). We compared sRAGE in AMAN between anti-GM1 antibody-positive and anti-GM1 antibody-negative cases. We found there was no difference between the two groups (p = 0.203). In addition, we compared sRAGE in AMAN between anti-GQ1b antibody-positive and anti-GQ1b antibody-negative cases. We similarly found there was no difference between groups (p = 0.894). CSF sRAGE levels were below the minimum detectable level in both patients with GBS and ONNDs.

### Increased serum HMGB1, TNF-α, and IL-6 levels in GBS

**Figure 3:**
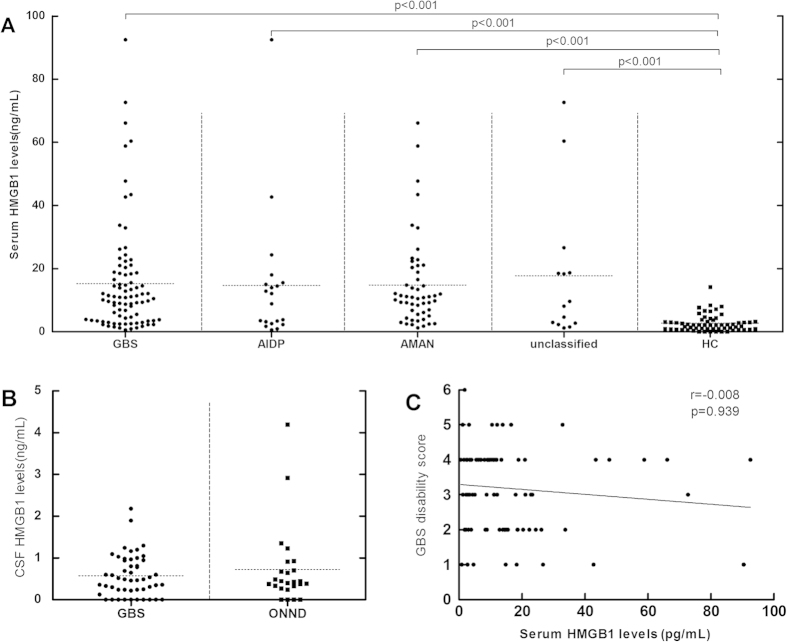
Serum and cerebrospinal fluid (CSF) high mobility group box 1 (HMGB1) levels in patients with Guillain-Barré syndrome (GBS). (**A**) Serum HMGB1 levels in 86 patients with GBS and 60 healthy controls. (**B**) CSF HMGB1 levels in patients with GBS and 60 healthy controls. (**C**) The relationship between serum HMGB1 levels and GBS disability score in GBS.

We determined serum and CSF HMGB1 levels to investigate whether differences in HMGB1 levels might be relevant to GBS. Serum HMGB1 levels in patients with GBS were significantly higher than controls (p < 0.001, Fig. 3A). Across the different subtypes of GBS, serum HMGB1 levels in patients with AMAN were similar to their AIDP (p = 0.382) and unclassified counterparts (p = 0.578). HMGB1 levels in all three subtypes were significantly higher than in control subjects (p < 0.001 for each subtype, Fig. 3A). We compared HMGB1 levels between anti-GM1 antibody-positive and anti-GM1 antibody-negative AMAN cases, and found no difference between groups (p = 0.123). Similarly, we found there was no difference in HMGB1 levels between anti-GQ1b antibody-positive and anti-GQ1b antibody-negative AMAN cases (p = 0.796). There was no significant difference in CSF HMGB1 levels between patients with GBS and the ONND patient group (p = 0.969, Fig. 3B).

**Figure 4:**
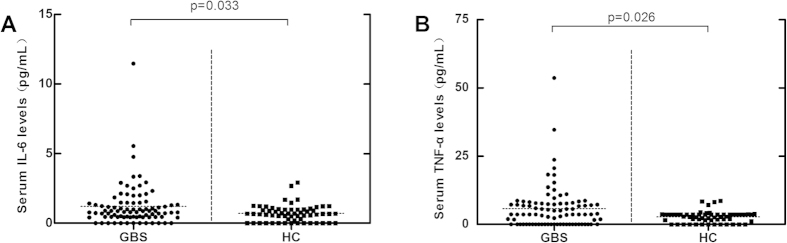
Serum IL-6 and TNF-α levels in patients with Guillain-Barré syndrome (GBS). (**A**) Serum IL-6 levels in patients with GBS and 60 healthy controls. (**B**) Serum TNF-α levels in patients with GBS and 60 healthy controls.

Serum HMGB1 levels were further analysed in the 13 patients with GBS at different points of the course of their disease and treatment schedule. We found that serum HMGB1 levels were significantly decreased in patients after treatment (p = 0.033, Fig. 2B), while in plateau phase serum HMGB1 levels were still decreased significantly (p = 0.028, Fig. 2B). There was no significant difference in sRAGE levels between patients at plateau stage and healthy controls (p = 0.241, Fig. 2B). As HMGB1 can induce IL-6 and TNF-α release from macrophage-like cells to enhance immune responses, we analysed serum IL-6 and TNF-α levels as well. Compared to control subjects, patients with GBS had higher IL-6 (p = 0.033, Fig. 4A) and TNF-α levels (p = 0.026, Fig. 4B).

To analyse the associations between sRAGE and HMGB1, and between HMGB1 with IL-6 and TNF-α, we investigated the percentage of patients with GBS who had both low sRAGE and high HMGB1. Normal levels were defined as the average serum levels observed in healthy controls according to the percentile method. If the detected serum levels was lower than P_5_, this value was considered lower than the normal level. If the detected serum levels was higherr than P_95_, this value was considered higher than the normal level. The percentage of patients with GBS who had both low sRAGE and high HMGB1 was 38.37%. The percentage of patients with AMAN who had both low sRAGE and high HMGB1 was 55.77%. The percentage of patients with higher HMGB1 serum levels who also displayed higher IL- 6 or TNF-α serum levels was 64.28%.

### Correlation between serum sRAGE or HMGB1 levels and disease severity

**Figure 5:**
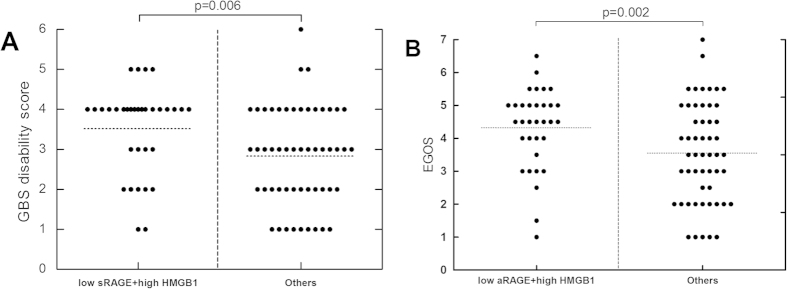
Guillain-Barré syndrome (GBS) disability scores and Erasmus GBS outcome scores (EGOS) in GBS patients with both low soluble receptor for advanced glycation end products (sRAGE) and high high mobility group box 1 (HMGB1) levels. (**A**) GBS disability scores in patients with GBS with both low sRAGE and high HMGB1 levels. (**B**) EGOS in patients with GBS with both low sRAGE and high HMGB1 levels.

To investigate the relationships between sRAGE and HMGB1 with disease severity, we performed correlation analyses between serum sRAGE levels and GBS disability score, and between HMGB1 levels and GBS disability score. We observed a significant inverse correlation between serum sRAGE levels and GBS disability score in the AMAN subtype (r = −0.445, p < 0.001, Fig. 1B). However, no significant correlations between HMGB1 levels and GBS disability score were found in patients with GBS overall (r = −0.008, p = 0.939, Fig. 3C). Moreover, we found that GBS disability scores in GBS patients with both low sRAGE and high HMGB1 levels were significantly higher than in all other patient groups studied (p = 0.006, Fig. 5A).

### Correlation between serum sRAGE or HMGB1 levels and GBS outcome

We performed correlation analyses between serum sRAGE and HMGB1 with EGOS to investigate whether sRAGE or HMGB1 level correlates with GBS outcome. We found a significant inverse correlation between serum sRAGE and EGOS in the AMAN subtype (r = −0.340, p = 0.014, Fig. 1C). There was not a significant correlation between serum HMGB1 and EGOS in GBS overall (r = −0.043, p = 0.693). We also found that EGOS in GBS patients with both low sRAGE and high HMGB1 levels were significantly higher than in other patients in the study (p = 0.002, Fig. 5B).

## Discussion

Our results showed that serum levels of sRAGE were significantly decreased only in the AMAN subtype of GBS. These findings suggest that lower serum sRAGE levels may be involved in the pathogenesis of inflammation in AMAN, but not AIDP, and therefore different immunological mechanisms may underlie AMAN and AIDP pathogenesis. In contrast, HMGB1 was increased across GBS subtypes, indicating that there may also be common mechanisms between subtypes as well.

Currently, the role of sRAGE in the process of inflammation is unclear, but it may operate by regulating mRAGE activity. mRAGE and its associated ligands regulate such diverse processes as inflammation, oxidative stress, neurodegeneration, promotion of neurite outgrowth, cell survival, and neuronal differentiation^25^,^26^. Interactions between mRAGE and its ligands are implicated in the pathogenesis of various disorders, such as neurodegenerative, inflammatory, and autoimmune disorders^27^,^28^,^29^. Notably, mRAGE has been found to be present not only in infiltrating mononuclear phagocytes, but also in neurons after injury during the inflammatory phases^10^,^11^,^30^. Several studies of animal models have reported that administration of recombinant sRAGE can block the mRAGE signalling pathway. Intravenously administered sRAGE acts as a decoy receptor to capture and hinder circulating RAGE ligands such as HMGB1 to protect against tissue damage^31^,^32^. Considering that the pathological manifestation of AMAN is mainly axonal impairment in peripheral nerves, we hypothesize that lower sRAGE levels in patients with AMAN may facilitate HMGB1 binding to mRAGE in neurons, thus enhancing RAGE signalling and the pathogenesis of AMAN. Significant inverse correlations between serum sRAGE levels and disease severity, and between serum sRAGE and GBS outcome in patients with AMAN were found in our data. These findings support the potential role of the RAGE axis in the clinical pathology of AMAN.

As the major RAGE ligand, HMGB1 interacts with mRAGE in monocytes, macrophages, and other cell types to enhance the inflammatory immune response. We found that serum HMGB1 levels were significantly higher in patients with GBS, irrespective of GBS phenotype, suggesting that serum HMGB1 was involved in inflammation in all subtypes of GBS. Consistent with previous studies, we also observed elevated serum TNF-α and IL-6 levels in patients with GBS^33^,^34^. Previous experimental studies have found that TNF-α has toxic effects on myelin, Schwann cells, and endothelial cells, and may be involved in the pathogenesis of demyelination and the breakdown of the blood-nerve barrier in autoimmune neuropathies^35^,^36^. Considering these roles for IL-6 and TNF-α in immunoinflammation and the pathology of GBS, induction of these cytokines by HMGB1 may be involved in the inflammatory processes underlying GBS pathogenesis.

A previous study found an increase in the CSF HMGB1 levels in patients with MS and NMO, which may have been related to CNS inflammation^12^. In addition, several studies have reported the breakdown of the blood-brain/nerve barrier in GBS, and the presence of CNS inflammation^37^,^38^. In contrast, we did not detect sRAGE or observe abnormal HMGB1 levels in the CSF in our study. Our results therefore seem to indicate that CNS inflammation in GBS, especially in AMAN, may be mild. However, the mechanistic reason for these apparently contradictory findings is unclear.

Finally, increased sRAGE levels and decreased HMGB1 levels were observed in patients with GBS after treatment, while sRAGE levels and HMGB1 levels appear to be normal in plateau phase. This question strongly warrants further inquiry, as recent studies have reported that administration of recombinant sRAGE or anti-HMGB1 monoclonal antibodies have therapeutic benefit for several diseases, including acute ischemic stroke for both therapies, autosomal dominant polycystic kidney disease in the case of recombinant RAGE, and sepsis and autoimmune myocarditis in the case of HMGB1 monoclonal antibody therapy^39^,^40^. Several studies have demonstrated that administration of recombinant sRAGE effectively protects brain tissues in a mouse model of stoke from injury and primary neurons in culture from cell death via a mechanism involving reduced activation of c-Jun amino-terminal kinase and NF-κB^39^,^41^. Administration of recombinant sRAGE in a mouse model of autosomal dominant polycystic kidney disease reduces cysts and promotes enhanced renal function, inhibition of cell proliferation, inflammation, and fibrosis^40^. Similarly, anti-HMGB1 monoclonal antibody intervention has been reported to reduce IL-6 and TNF-α release from macrophage-like cells, to attenuate lethality in an established sepsis^42^. Considering the results of the present study together with these previous findings, we hypothesize that anti-HMGB1 monoclonal antibody therapy may be effective in treating GBS generally, while sRAGE may be a promising target for treating the AMAN subtype specifically.

In conclusion, our study showed that serum sRAGE levels were lower only in patients with the AMAN subtype of GBS, while elevated serum HMGB1 levels was observed in all subtypes. Lower sRAGE and higher HMGB1 levels may be related to the robust autoimmune response that underlies GBS, perhaps through increasing the release of inflammatory cytokines. Clinically, serum sRAGE may be a potential biomarker for disease severity in the AMAN subtype, while recombinant sRAGE and anti-HMGB1 interventions may represent promising therapies for treating GBS.

## Materials and Methods

### Subjects

A total of 86 consecutive patients with GBS were recruited from the Neurology Department of Tianjin Medical University General Hospital from January 2011 to November 2015. The diagnosis of GBS fulfilled the 1990 Asbury and Cornblath criteria^43^. All subjects were negative for hypokalaemia, hyperkalaemia, porphyria, connective tissue disorder, and other neurological disorders. According to Ho’s criteria^3^, the patient cohort was divided into three subtypes based on their electrophysiological data: 20 patients were classified with AIDP; 52 patients with AMAN, including 32 patients with AMSAN; and 14 patients with an unclassified subtype. Sixty healthy sex- and age-matched controls were recruited from the health centre of our hospital for this study. All the research procedures were approved by the ethics committee of Tianjin Medical University General Hospital, and all the participants provided written informed consent prior to participation. All the research procedures were conducted in accordance to guidelines for experiments of the ethics committee of Tianjin Medical University General Hospital.

### Data and sample collection

The data for demographic features, history of disease, preceding infection, GBS disability score, Erasmus GBS outcome score (EGOS), and anti-GM1/GQ1b antibody status, as well as CSF, electrophysiology, and therapy data, were obtained from our GBS database. The severity of GBS was evaluated based on the GBS disability score and the EGOS score was used to predict outcome at 6 months^44^,^45^. Both the GBS disability score and EGOS were assessed at 2 weeks after entry. Serum anti-GM1 and anti-GQ1b antibodies were detected using an enzyme-linked immunosorbent assay (ELISA) kit (CUSABIO, Wuhan Hua Mei, China). Electrophysiological examinations were performed at 14.3 ± 4.6 days from disease onset.

Eighty-six serum samples were collected during acute exacerbations but prior to treatment onset. Only 50 CSF samples matched with serum samples were obtained after lumbar puncture, of which 17 CSF samples were collected within 2 days after treatment with intravenous immunoglobulin. We also collected CSF samples of 25 patients with other non-inflammatory neurological disorders (ONNDs) as controls, comprising: 7 subacute combined degeneration of the spinal cord cases, 10 motor neuron disease cases, 1 alcoholic peripheral neuropathy case, 2 Wernicke-Korsakoff syndrome cases, 2 Friedreich’s ataxia cases, 1 intracranial hypotension syndrome case, and 2 muscular dystrophy cases. Clinical data were recorded at the time of blood collection. All samples were stored at −80 °C until analysis. In addition, thirteen serum samples from the enrolled patients with GBS were collected when clinical status improved after intravenous immunoglobulin therapy at a dose of 0.4 g/Kg/day for 5 consecutive days, such that the median interval of the serum sampling collection was 14 days from onset day (range, 9–29 days). Moreover, there were six serum samples, among the 13 serum samples, that were collected in plateau phase (range, 62–91 days from onset day).

### Measurement of serum and CSF sRAGE

We examined serum sRAGE levels using a human RAGE ELISA kit (R&D Systems, Minneapolis, MN, USA) according to the manufacturer instructions. We also determined CSF sRAGE levels in patients with GBS and ONNDs using the same ELISA kit. Briefly, serum samples were diluted 2 times, and CSF samples were used undiluted. Samples were then added to wells coated with monoclonal antibodies for human RAGE. Next, they were incubated with a polyclonal anti-human RAGE antibody conjugated to horseradish peroxidase. The absorbance values of the corresponding substrate were detected at 450 nm. sRAGE levels were calculated with reference to a standard curve. The lowest detectable level was 4.21 pg/ml.

### Measurements of serum and CSF HMGB1 levels

Serum HMGB1 levels were detected in patients with GBS and healthy controls using an HMGB1 ELISA Kit II (Shino-Test Corporation, Tokyo, Japan), according to the manufacturer’s instructions. The CSF HMGB1 levels in patients with GBS and ONNDs were also determined with the same ELISA kit. In brief, 110 μl of diluted samples (10 μl of serum and 100 μl of diluent, 50 μl of CSF and 60 μl of diluent) was added to an ELISA plate coated with anti-HMGB1 antibody for 24 h at 37 °C. After washing, we added 100 μl of detection antibody to each well and incubated the preparations for 2 h. Next, 100 μl of colour reagent was added to each well and the solution was incubated for 30 min. Finally, 100 μl of stop solution was added. The absorbance values were measured at 450 nm. HMGB1 levels were calculated with reference to a standard curve. The lowest detectable level was 0.2 pg/ml. If the detected value is less than lowest detectable level, the determined level is zero.

### Measurements of serum IL-6 and TNF-α levels

We used a human IL-6 or TNF**-**α high sensitivity ELISA kit (eBioscience, San Diego, CA, USA) to detect IL-6 and TNF-α levels according to the manufacturer’s instructions. Briefly, serum samples diluted 5 times were added to wells coated with monoclonal anti-human IL-6 or TNF-α antibody. Next, they were incubated with a biotin-labelled secondary polyclonal anti-human IL-6 or TNF-α antibody for 2 h. After washing, streptavidin-horseradish peroxidase (HRP) conjugate was added for 1 h. Then, the amplification solution I was added for 15 minutes. Finally, the amplification solution II was added. The absorbance values of the chromogenic substrate were detected at 450 nm. IL-6 and TNF-α levels were calculated with reference to a standard curve. The lowest detectable level of IL-6 and TNF-α was 0.08 pg/ml and 0.31 pg/ml, respectively. If the detected value was less than the lowest detectable level, the level was considered to be zero.

### Statistical analyses

Where appropriate, differences between groups were performed for continuous variables using unpaired t-tests, Mann Whitney U-tests, or Wilcoxon signed-rank tests. For the categorical variables, Chi-square tests or Fisher’s Exact test was used. The relationship between sRAGE, HMGB1, and EGOS was evaluated by Spearman’s rank correlation analysis. A P value of 0.05 or less was considered significant for all statistical analyses. All statistical analyses and graphs were performed using GraphPad PRISM 5 (Graph Pad Software Inc., San Diego, CA, USA).

## Additional Information

**How to cite this article**: Zhang, D.-Q. *et al.* Reduced soluble RAGE is associated with disease severity of axonal Guillain-Barré syndrome. *Sci. Rep.* **6**, 21890; doi: 10.1038/srep21890 (2016).

## Figures and Tables

**Table 1:** Demographic and clinical features of subjects.

Parameters	AMAN (n = 52)	AIDP (n = 20)	Unclassified (n = 14)
Age at disease onset (years)	46.23 ± 15.74 (17–75)	41.95 ± 20.30 (17–76)	43.21 ± 15.73 (24–75)
Gender (F/M)	23/29	7/13	8/6
Symptoms preceding infection	27	8	7
Diarrhea	14	3	3
Upper respiratory tract infection	13	5	4
GBS disability score at 2 weeks after entry	3.67 ± 0.86	2.55 ± 1.23**	1.79 ± 0.98**
Erasmus GBS outcome score	4.52 ± 1.00	3.15 ± 1.61**	2.14 ± 1.09**
Anti-ganglioside antibody (%)			
Anti-GM1-positive	36.96	16.67	7.14
Anti-GQ1b-positive	8.70	5.00	42.86
WBC in CSF (10^6^/L)	2 (0–10)	2 (0–10)	2 (0–6)
Protein in CSF (g/L)	1.16 ± 0.66	0.72 ± 0.35*	0.72 ± 0.37*

Data are expressed as mean ± SD, Mann–Whitney U tests were used for statistical analysis. *Statistically significant in comparison with AMAN, *P < 0.05, **P < 0.001. AMAN, acute motor axonal neuropathy; AIDP, acute inflammatory demyelinating polyneuropathy; CSF, cerebrospinal fluid; GBS, Guillain-Barré syndrome.
